# Enterovesical Fistula Revealing Crohn’s Disease: A Case Report

**DOI:** 10.7759/cureus.56690

**Published:** 2024-03-22

**Authors:** Touil Mohammed Amine, Mohamed Mokhtari, Hammou El Farhaoui, Anouar El Moudane, Ali Barki

**Affiliations:** 1 Urology, Mohammed VI University Hospital Center, Oujda, MAR; 2 Urology, Mohammed VI University Hospital, Oujda, MAR; 3 Urology, Mohammed I University, Oujda, MAR

**Keywords:** pseudotumor, colite, crohn, entérovésical, fistula

## Abstract

Crohn's disease is an inflammatory bowel disease of unknown etiology. It is characterized by intra- and extra-intestinal complications. It is a frequent cause of uroenteric fistulas. They are mostly symptomatic and occur after several years of the evolution of Crohn's disease. The pneumaturia and fecaluria are the most significant symptoms for their presence. They are usually poorly tolerated and require surgical treatment.

We report the case of an enterovesical fistula revealing Crohn's disease during endoscopic resection of a bladder pseudotumor in a generally impaired patient.

Crohn’s disease should be evocated when histology is not relevant for a bladder pseudotumor or a rectovesical fistula. The discovery of an isolated bladder pseudotumor should suggest the diagnosis in the context of weight loss and chronic diarrhea. Enterovesical fistulas are uncommon but potentially dangerous complications of Crohn’s disease.

Abdominal CT scans and cystoscopy are the most commonly used diagnostic modalities. Surgical treatment seems to be unavoidable in most cases although medical treatment could also benefit a small cohort of patients.

## Introduction

Crohn's disease is a chronic inflammatory disease of the digestive tract that can affect any of its segments. Enterovesical fistulas secondary to this disease are rare. They are most often symptomatic and occur after several years of development. They are generally poorly tolerated and require surgical treatment.

We report the case of an enterovesical fistula diagnosed late during endoscopic resection of a pseudotumor of the bladder in a 78-year-old patient, all evolving in the context of alteration of the general state.

## Case presentation

RY, a 78-year-old male, non-smoker, presented in the emergency room for deterioration of his general condition with hematuria and melena of low abundance associated with a loss of 30 kg in three years. The patient's hemodynamic status was stable. Cytobacteriological examination of urine was negative. There was a biological inflammatory syndrome with anemia, hemoglobin at 4 g/dl, and positive urine cytology. Ultrasound revealed an unusual-looking vegetative lesion on the right side of the bladder. Cystoscopy showed an inflammatory-looking polyp at the level of the bladder dome with the appearance of a bladder fistula. The cystography objectified the presence of an enterovesical fistula. He underwent endoscopic resection of the bladder polyp followed by bladder catheterization. Histological examination of the resection showed non-specific inflammatory lesions.

**Figure 1:**
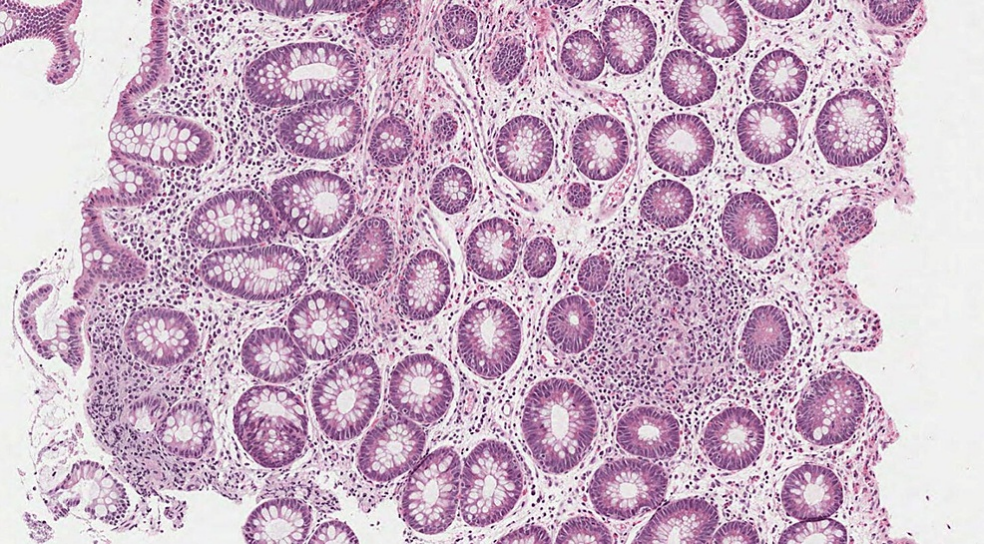
Photomicrograph showing a colic mucosa containing distorted crypts and a moderate inflammatory infiltrate, with the presence of an epithelioid granuloma

After the patient was transfused with three packed red blood cells, the colonoscopy carried out in the emergency objectified the presence of a colo-bladder fistula without other localizations, and the anatomopathology confirmed the presence of the epithelioid cells without caseous necrosis (Figure 1).

**Figure 2:**
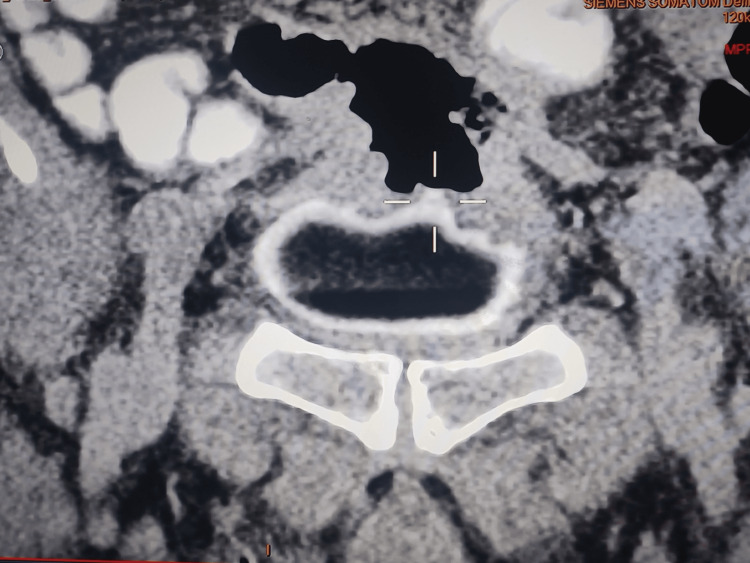
Abdominal-pelvic scanner objectifying the enterovesical fistula

An abdominopelvic scan was performed showing the colovesical fistula (Figure 2).

The patient suffered a hemorrhagic shock and was transferred to intensive care for further treatment, but he, unfortunately, died before completing his medical and surgical treatment.

## Discussion

Fistulas are rare complications (colovesical in 70% of cases, ileovesical, and rectovesical). They are secondary to pelvic inflammatory processes (diverticulitis, sigmoiditis, tuberculosis, or radiotherapy) or neoplastic (rectosigmoid cancer) [1]. They are more rarely of iatrogenic origin or secondary to urogenital lymphoma.

These fistulas often affect the bladder dome with male predominance due to the absence of uterine interposition. Patients with an enterovesical fistula typically present with lower urinary tract urinary disorders, which include pneumaturia (the most common symptom present in 50-70% of cases), fecaluria (reported in up to 51% of cases), pollakiuria, hypogastralgia, repeated urinary tract infections, and hematuria. In patients with Crohn's disease fistula, transit disorders, abdominal pain, abdominal mass, and abscesses are commonly found [1]. The diagnosis of enterovesical fistulas is confirmed by highlighting the fistulous path. No additional examination is particularly decisive, with the diagnosis being usually made by a set of clinical and paraclinical arguments. Radiology can be inconclusive in many situations, so barium enema only shows a vesico-sigmoid fistula in 25% of cases. This examination can be supplemented by a colonoscopy, which could be hampered by inflammation, making any biopsy random [2]. These two assessments remain essential to eliminate a recto-colic malignant cause. Abdominal CT scan has become the essential diagnostic examination coupled with digestive opacification by water-soluble or air enema. The most constant sign is an intravesical air bubble (gaseous crescent). Communication is rarely demonstrated (20-40% of cases). The scanner can eliminate a pelvic, bladder, rectal, or gynecological tumor. It also allows local assessment to plan a surgical strategy [3]. Magnetic resonance imaging allows accurate representation of fistulas without the need for direct opacification as in CT. Its use in colovesical fistulas is well-established, and its sensitivity and specificity reach 100% [4].

Cystoscopy retains an important place in the assessment of vesico-sigmoid fistulae, although it is unremarkable in a quarter of cases [3]. The most typical appearance is a pseudo-tumor area around the fistulous orifice. A gas bubble may exit through the fistula. The appearance is not very specific and can take the form of an infiltrating tumor. The localization sits more readily in the dome and on the posterior face of the bladder.

A vesico-enteral fistula is not a surgical emergency. It remains difficult to treat in patients with Crohn's disease and often requires a multidisciplinary approach.

The medical treatment of Crohn's disease has evolved rapidly since the introduction of anti-tumor necrosis factor (TNF) biologics such as infliximab. Immunosuppressive medical therapies appeared less effective compared to anti-TNF treatments. The authors argued that the mere presence of a fistula is not an indication for surgery, pointing out that medical therapy can successfully induce long-term remission [5]. In patients in poor general condition or with a well-tolerated vesico-enteral fistula, management consists of prolonged bladder catheterization in addition to medical treatment. For the other cases, a segmental colectomy is performed with the closure of the bladder breach, possibly an epipolar interposition, and bladder drainage for 10 days. The restoration of continuity will, in principle, be carried out at the same time if local conditions (no peritoneal abscess collection or digestive preparation) and general conditions (good general condition) allow it [5].

## Conclusions

An enterovesical fistula is a rare complication of Crohn's disease. The diagnosis is based on a combination of clinical, radiological, endoscopic, and pathological arguments. Surgical treatment should be performed after failure of medical treatment, in patients in good general condition, and in whom the symptoms are disabling.
